# Understanding the Origin of Metal Gate Work Function Shift and Its Impact on Erase Performance in 3D NAND Flash Memories

**DOI:** 10.3390/mi12091084

**Published:** 2021-09-08

**Authors:** Sivaramakrishnan Ramesh, Arjun Ajaykumar, Lars-Åke Ragnarsson, Laurent Breuil, Gabriel Khalil El Hajjam, Ben Kaczer, Attilio Belmonte, Laura Nyns, Jean-Philippe Soulié, Geert Van den bosch, Maarten Rosmeulen

**Affiliations:** IMEC, Kapeldreef 75, B-3001 Leuven, Belgium; ARJUN007@e.ntu.edu.sg (A.A.); Lars-Ake.Ragnarsson@imec.be (L.-Å.R.); Laurent.Breuil@imec.be (L.B.); k.elhajjam@gmail.com (G.K.E.H.); Ben.Kaczer@imec.be (B.K.); Attilio.Belmonte@imec.be (A.B.); Laura.Nyns@imec.be (L.N.); Jean-Philippe.Soulie@imec.be (J.-P.S.); Geert.VandenBosch@imec.be (G.V.d.b.); Maarten.Rosmeulen@imec.be (M.R.)

**Keywords:** work function, effective work function, dipole, metal gate, high-k, SiO_2_, interfacial reaction, MHONOS, erase performance, 3D NAND flash memory

## Abstract

We studied the metal gate work function of different metal electrode and high-k dielectric combinations by monitoring the flat band voltage shift with dielectric thicknesses using capacitance–voltage measurements. We investigated the impact of different thermal treatments on the work function and linked any shift in the work function, leading to an effective work function, to the dipole formation at the metal/high-k and/or high-k/SiO_2_ interface. We corroborated the findings with the erase performance of metal/high-k/ONO/Si (MHONOS) capacitors that are identical to the gate stack in three-dimensional (3D) NAND flash. We demonstrate that though the work function extraction is convoluted by the dipole formation, the erase performance is not significantly affected by it.

## 1. Introduction

When it comes to low-cost and large density non-volatile memory, three-dimensional (3D) NAND flash memory technology is the industry standard [1,2]. The memory stack used in 3D NAND is inspired by a typical SONOS memory cell, which allows easy vertical integration and is addressed by horizontal word lines (WL). To improve the bit density, the number of cells in the vertical 3D NAND string is increased. This requires the stacking of many WLs, which need to be as thin as possible to limit the total height and mechanical stress of the structure [3]. Tungsten (W) metal-based WL is currently being used by the industry. However, novel materials with lower resistivity are being considered as future candidates to reduce the high resistive-capacitive (RC) delay that results as a consequence of WL thinning and continued stacking of the WLs (i.e., downscaling the metal thickness) in the vertical direction.

**Figure 1:**
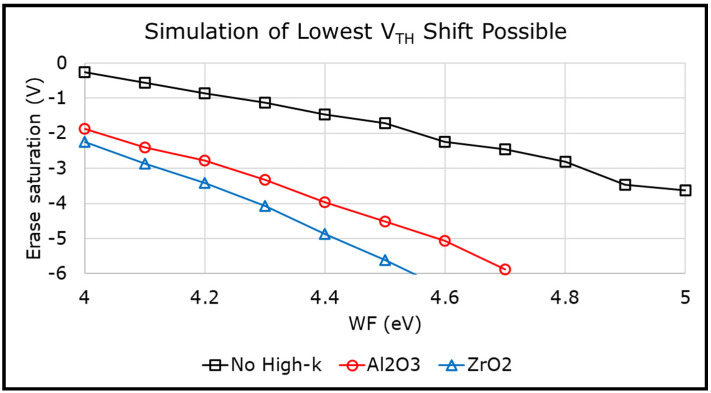
Simulations of erase saturation levels in a memory stack without high-k liner, or with 2 nm Al_2_O_3_ or ZrO_2_. Addition of a high-k liner shows more benefit than (work function) WF.

Moreover, the WL metal can act as an enabler to improve the 3D NAND erase operation. It was shown that high work function metals, such as TiN and Ru, can delay the electron injection from the gate (i.e., electrons tunneling from the gate into the charge-trap layer), thereby improving the erase window [4]. It has also been demonstrated [5] that when a metal gate is used in combination with a thin high-k liner, such as Al_2_O_3_, HfO_2_, or ZrO_2_ (i.e., a Metal/High-k/ONO/Si (MHONOS) structure), the erase performance can be further improved. Figure 1 plots the erase saturation levels (lowest possible threshold voltage, *V_TH_*, shift achievable) for different scenarios, with and without a high-k liner, as simulated using our in-house developed 1D simulator [6]. The high-k liner helps to lower the injecting field for the electrons at the gate, and even proves to have a larger impact than the metal work function (WF). The erase is found to be penalized when the MHONOS stack is treated with a high thermal budget [3]. To thoroughly investigate the WL metal and high-k liner combination, and its effect on erase operation, metal work function extraction experiments have been proposed and studied in this work.

WF analysis of metal gate electrodes on high-k dielectrics, by monitoring flat-band voltage, *V_FB_* (or threshold voltage, *V_TH_*), have been demonstrated in the literature [7,8,9,10,11,12]. The studies report an undesirable shift in the *V_FB_* (or *V_TH_*) of metal-oxide-semiconductor (MOS) devices. The origins are unclear, leading to an effective work function (eWF) for the metal, different from the bulk values. Some reports in the literature attribute this shift to Fermi level pinning (FLP) caused either by metal-induced gap states [13,14,15] or charged defects/oxygen transfers, at the metal/high-k interface [12,16,17]. Dipole formation at the high-k/SiO_2_ interface due to oxygen vacancies [18,19], and/or the energy offsets between the high-k and SiO_2_ [20], have also been suggested in the literature as possible root causes for an eWF. Though, these studies suggest a notable dependence of eWF on the choice of high-k used, other process parameters such as gate electrode deposition and annealing conditions have been found to affect the eWF in a significant way as well [21].

In this paper, we investigate the change in WF (i.e., eWF) of metal electrodes deposited on high-k dielectrics. Based on the process conditions used, we evidence it to either the interfacial reactions at the WL-to-high-k contact or between the high-k and the oxide. The aim of this work is to understand the origins and consequences of WF shifts based on process conditions within the context of 3D NAND flash memory devices. Therefore, we also analyze various MHONOS stacks containing Al_2_O_3_, ZrO_2_, HfO_2_ high-k liners and TiN, Ru, Mo as gate metal, and corroborate the eWF with the erase performance of these stacks.

## 2. Materials and Methods

Capacitors with and without the charge trap layer were fabricated on 300 mm p-doped Si (100) wafers for erase analysis and WF extraction, respectively.

### 2.1. Work Function Extraction Methodology

**Figure 2:**
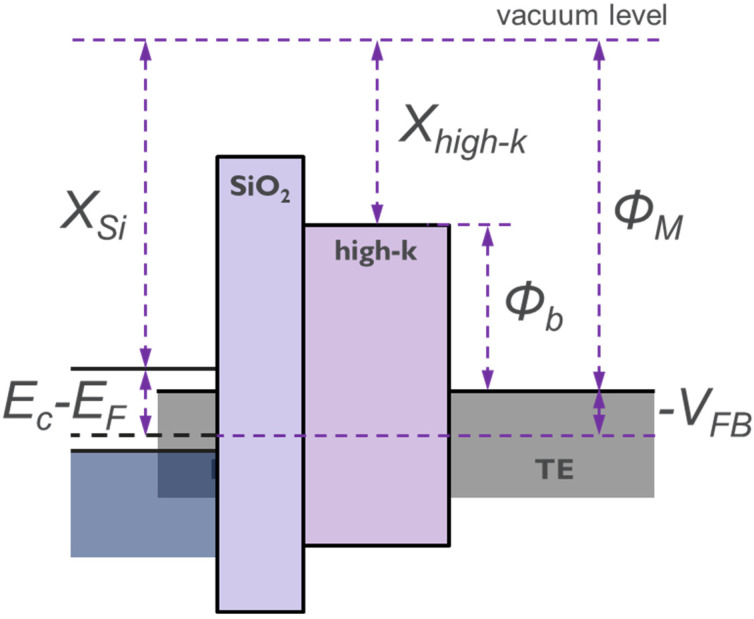
Schematic of the energy band diagram of a metal-insulator-semiconductor (MIS) capacitor.

The WF of a metal on high-k is determined by extracting *V_FB_* from capacitance–voltage (CV) measurements on a metal-insulator-semiconductor (MIS) structure [22]. The schematic in Figure 2 shows the energy band diagram of an MIS structure. From this, we note that the metal work function can be expressed as follows

(1) $$\Phi_{M}=V_{FB}+\chi_{Si}+\left[E_{C}-E_{F}\right],$$

where *Φ_M_* is metal work function, *V_FB_* is flat-band voltage computed from CV measurements, *χ_Si_* is electron affinity of Si substrate, *E_C_* and *E_F_* are the conduction band minima and fermi level.

However, the charges present in the bulk and at the interfaces of the oxides [23] can affect the *V_FB_* as follows

(2) $$\Delta V_{FB}=\int_{0}^{t_{ox}}\frac{\rho \left(z\right)\left(t_{ox}-z\right)}{\varepsilon \left(z\right)\varepsilon_{0}}dz,$$

**Figure 3:**
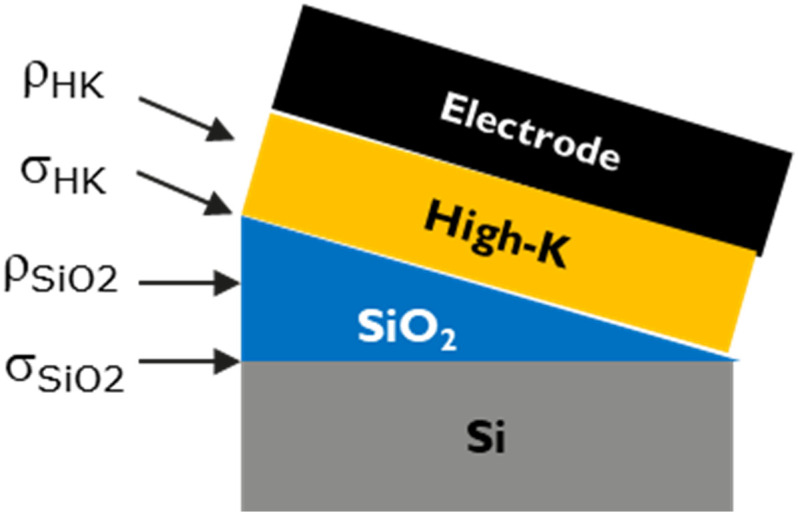
Schematic of MIS capacitor with slant etch for SiO_2_. Corresponding oxide charge densities are indicated.

From the above equation, it is clear that the effect of these oxide charges can be cancelled out by extracting the *V_FB_* at zero oxide thickness. This calls for variations in SiO_2_ and high-k thicknesses. With the help of a slant etch technique, the thickness of SiO_2_ was varied across the wafer as shown in schematic in Figure 3. For each electrode, a set of 3 wafers with different high-k thicknesses (typically 3 nm, 5 nm, 7 nm) was used to provide enough variation and extract the WF conveniently. Typical CV measurements and *V_FB_* extraction procedure are discussed in Appendix A.

The impact of oxide charges on *V_FB_* can be mathematically expressed in terms of equivalent oxide thickness (EOT) and the corresponding charge densities as follows [24]

(3) $$V_{FB}=\Phi_{MS}+q\cdot \rho_{HK}\cdot \varepsilon_{HK}\cdot \frac{EOT_{HK}^{2}}{2\cdot \varepsilon_{ox}^{2}\cdot \varepsilon_{o}}+q\cdot \sigma_{HK}\cdot \frac{EOT_{HK}}{\varepsilon_{ox}\cdot \varepsilon_{o}}+q\cdot \rho_{SiO_{2}}\cdot \frac{0.5\cdot T_{SiO_{2}}^{2}+\left(\frac{\varepsilon_{HK}}{\varepsilon_{ox}}\right)\cdot T_{SiO_{2}}\cdot EOT_{HK}}{\varepsilon_{ox}\cdot \varepsilon_{o}}+q\cdot \sigma_{SiO_{2}}\cdot \frac{EOT_{total}}{\varepsilon_{ox}\cdot \varepsilon_{o}},$$

where *q* is the electron charge, *ρ_HK_* and *σ_HK_* are the bulk and interface charge densities of high-k dielectric, respectively. The terms *ρ_SIO__2_* and *σ_SIO__2_* are the corresponding bulk and interface charge densities of SiO_2_, respectively. *EOT_HK_*, *T_SiO_*_2_, and *EOT_total_* are the equivalent oxide thickness of high-k, thickness of SiO_2_, and both combined, respectively. The *EOT_total_* is in fact the measured EOT computed from the CV measurement of the MIS capacitors. The terms *ε_HK_*, *ε_ox_*, *ε_o_* are the relative permittivity of high-k, SiO_2_ and permittivity of free space, respectively. The *Φ_MS_* in the above equation, from which the metal WF is extracted, is later computed by extrapolating *V_FB_* at EOT (both high-k and SiO_2_) = 0.

**Figure 4:**
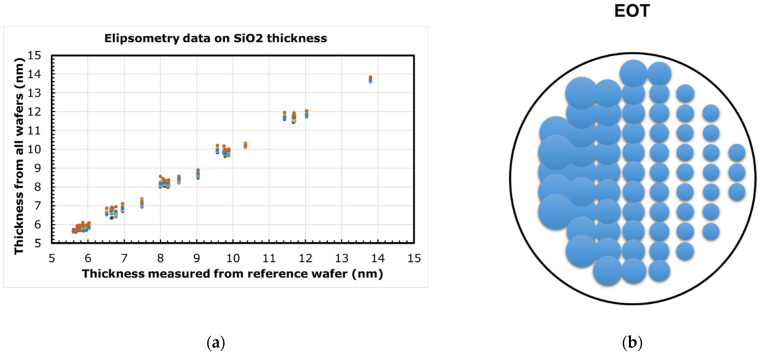
(**a**) Thickness of SiO_2_, after slant etch and plasma enhanced atomic layer deposition (PEALD) oxide deposition, measured across multiple wafers using ellipsometry; (**b**) Equivalent oxide thickness (EOT) computed from capacitance–voltage (CV) measurement. Bubble size represents EOT magnitude.

First, a 30 nm thick layer of high quality SiO_2_ was thermally grown at 900 °C. This was then etched back with a slant profile (as shown in Figure 3) by slowly immersing (at a constant rate) the wafer in a 1.9% hydrofluoric acid (HF) solution. The desired thickness range of SiO_2_ is obtained across the wafer by modifying the rate of immersion accordingly. A nominal thickness range of 3–12 nm was used in this work. Then, after the slant etch, a 3 nm plasma enhanced atomic layer deposition (PEALD) SiO_2_ was uniformly deposited at 300 °C, to mimic the blocking oxide in a 3D NAND device. Little wafer-to-wafer variations were observed in the oxide thickness, as measured by ellipsometry (see Figure 4a). The total EOT measured from CV will vary across the wafer due to the slant etch of thermal oxide, as shown in Figure 4b (bubble size represents magnitude of EOT).

**Figure 5:**
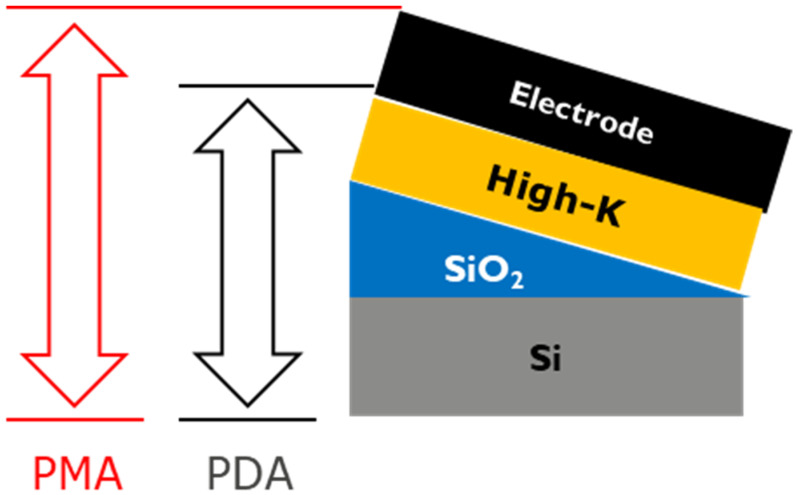
Schematic indicating different anneal types and the corresponding layers that received the process.

After this, high-k liners, such as Al_2_O_3_, ZrO_2_, and HfO_2_, were deposited at 300 °C to their desired thicknesses, using atomic layer deposition (ALD). Finally, 20 nm ALD Ru or ALD TiN or PVD Mo were then deposited as the gate electrode. In order to isolate the impact of thermal treatment on individual layers, a high temperature anneal (*T_anneal_*) was performed at different stages of the stack formation (as shown in Figure 5). For instance, some of the capacitors were subjected to a post metallization anneal (PMA) for 20 min at 750 °C in N_2_ ambient. A few others were subjected to a post high-k deposition anneal (PDA), where the entire stack sans the metal electrode received a thermal treatment for 1 min at 1050 °C for Al_2_O_3_-based stacks and 1 min at 750 °C for the rest, all in N_2_ ambient. All wafers received a final sintering anneal in 5 atm H_2_ ambient at 450 °C for 30 min.

CV measurements were performed on 70 × 70 μm^2^ capacitors at a frequency of 100 kHz. The parameters needed for the WF extraction, namely, *V_FB_*, the substrate doping concentration and the total EOT, EOT_total_, are estimated (see Appendix A) with the help of NCSU’s CVC model fitting software [25]. Based on the expression for *V_FB_* from Equation (3), we can express *V_FB_* as a second order polynomial equation in terms of the EOT, as the one below

(4) $$V_{FB}=\Phi_{MS}+a\cdot EOT_{HK}^{2}+b\cdot EOT_{HK}+p\cdot T_{SiO_{2}}^{2}+q\cdot T_{SiO_{2}},$$

where *a*, *b*, *p*, and *q* contain the charge densities of high-k and SiO_2_.

**Figure 6:**
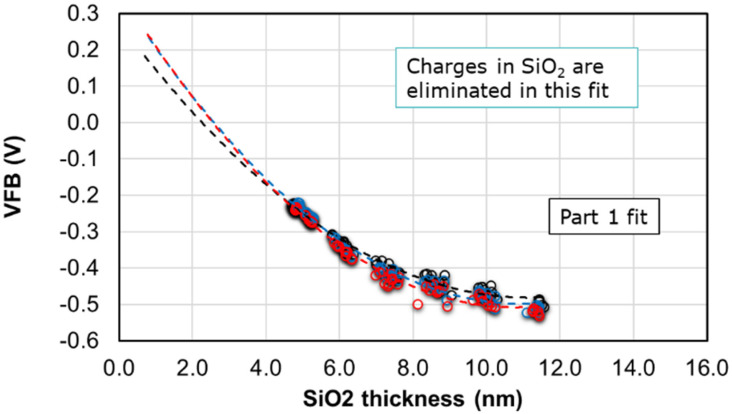
The *V_FB_* measured from CV is plotted as a function of SiO_2_ thickness. A second order fit is performed to isolate the terms p and q containing the charge densities in its bulk and interface.

From the above equation, we can first eliminate the effect of charges in SiO_2_ with a second order polynomial fit of the *V_FB_* with the thickness of SiO_2_, *T_SiO_*_2_. A sample fit is shown in Figure 6. The intercept from the first fit contains the polynomial equation with high-k EOT, EOT_HK_ and hence is used to eliminate the charges from high-k in a second fit.

As mentioned earlier, we have the *EOT_total_* of the stack as measured from CV. In order to get the *T_SiO_*_2_ to be used in the first fit, we make use of the ellipsometry data that was measured at preset locations across the wafer, after the slant etch and PEALD deposition. This data is then compared with corresponding dies for which the CV was measured. The difference between the measured *EOT_total_* and this ellipsometry data will give an estimate of the *EOT_HK_*.

**Figure 7:**
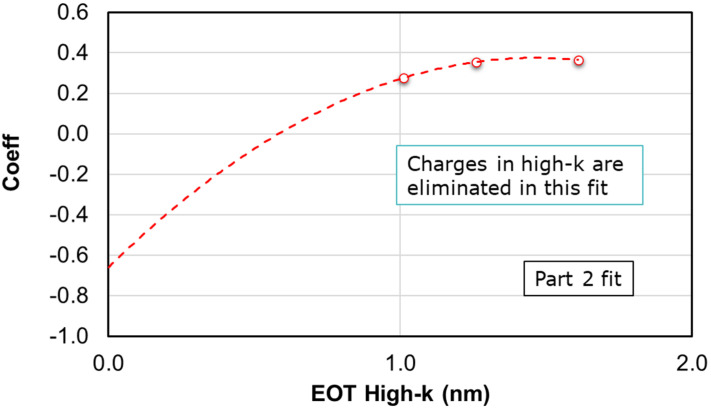
The intercepts from the part 1 fit are plotted as a function of high-k EOT. A second order fit is performed to extract the metal work function.

The three curves shown in Figure 6 represent the three wafers with three different high-k thicknesses needed for sufficient variation to eliminate the charges affecting the *V_FB_*. The corresponding intercept from the 2nd order fit of the above curves is then used in a second fit, as shown in Figure 7 below.

The intercepts vs. the EOT_HK_ will now help to eliminate the charges in high-k. The intercept from this second fit is the *Φ_MS_* from which the WF is computed using the formula

(5) $$WF=4.05+\Phi_{MS}+E_{C}-E_{F},$$

where $E_{C}-E_{F}\left(in\;eV\right)=1.12-0.0257*\ln\left(\frac{1.83E19}{median\;doping\;concentration\;in\;the\;substrate}\right).$

### 2.2. NAND Flash Erase Analysis

Incremental Step Pulse Erase (ISPE) characteristics were studied by monitoring the shift in *V_TH_* of MHONOS capacitors from their fresh state. The erase operation is divided into a number of steps with increasing amplitude (for a duration of 1 ms) in applied voltage and at the end of each of them a verify operation is applied to check the *V_TH_*. The amplitude and rate of change in *V_TH_* is considered as a measure of erase performance.

**Figure 8:**
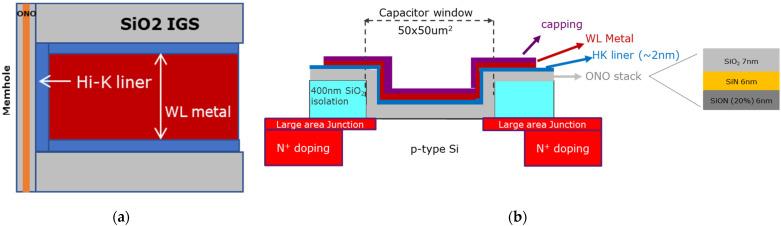
(**a**) Cross-section schematic of the memory gate stack in a vertical three-dimensional (3D) NAND device; (**b**) schematic of a planar test structure used in this work. The components of the gate stack are indicated in the figure.

Large MHONOS capacitors (50 × 50 μm^2^) were fabricated on 300 mm p-doped Si (100) wafers, as shown in Figure 8b. N^+^-doped rings were processed, surrounding the active area of the capacitors, to provide minority carriers for program operation. In a study reported elsewhere [3], we have demonstrated a 3D NAND test structure with 5 layers and showed that the memory characteristics of the stack (see Figure 8a) are qualitatively similar to that of the planar test structures that we typically use (see Figure 8b). Moreover, the gate stack deposited in this work mimics the one of 3D NAND in production [3,26] in terms of annealing processes and high-k/metal gate depositions performed. Therefore, we could fairly say that the results obtained from the planar capacitors in this work are relevant for 3D NAND flash memory devices.

**Figure 9:**
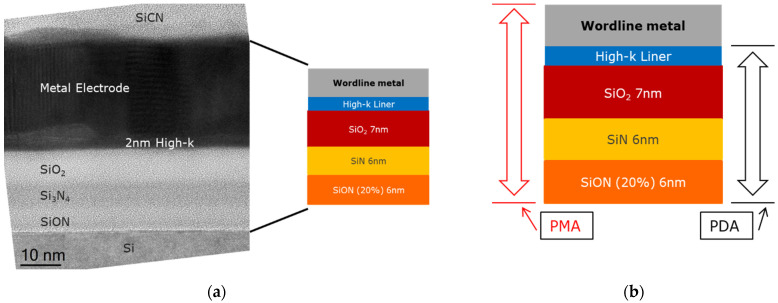
(**a**) Transmission electron microscope (TEM) image of a memory stack fabricated in this work; (**b**) different anneal types and the corresponding MHONOS layers that received the anneal.

The MHONO stack, as seen from the TEM image in Figure 9a, consists of a 6 nm SiON (with 20% N-to-O ratio) tunnel layer deposited using CVD at 780 °C, 6 nm LPCVD Si_3_N_4_ charge trap layer deposited at 690 °C, 7 nm PEALD SiO_2_ blocking oxide deposited at 300 °C, and 2 nm ALD Al_2_O_3_ or ZrO_2_ or HfO_2_ high-k liner deposited at 300 °C. A total of 20 nm ALD Ru or ALD TiN or PVD Mo were then deposited as the gate electrode (WL, wordline). Similar to the study of WF extraction, a post metallization anneal, PMA for 20 min at 750 °C in N_2_ ambient, and a post deposition anneal, PDA for 2 min at 1050 °C for Al_2_O_3_ based stacks and 1 min at 750 °C for the rest, all in N_2_ ambient, were performed for some of the capacitors (see Figure 9b). All wafers were subject to a final sintering anneal either in forming gas at 420 °C for 20 min or in 5 atm H_2_ ambient at 450 °C for 30 min. We may note that the sintering anneal has little influence on the final erase saturation levels.

## 3. Results and Discussion

**Figure 10:**
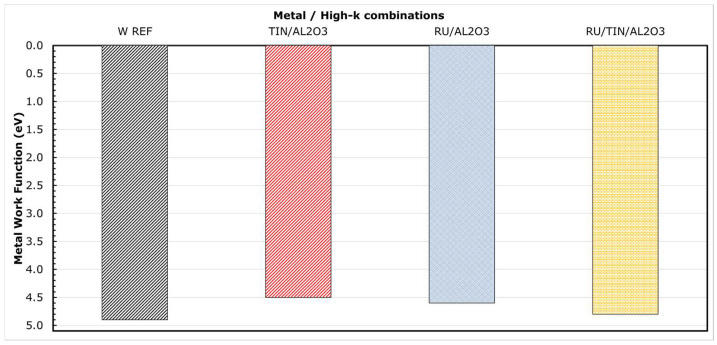
Metal work function listed for a few metal/high-k combinations from this work. No high temperature anneals were performed for these stacks.

The metal WF extracted in this work are listed as a histogram plot in Figure 10 for a few metal/high-k combinations. No high temperature anneals were performed for these splits. W Ref represents the CVD W/thin (3 nm) ALD TiN/Al_2_O_3_ liner stack similar to the one used currently in 3D NAND production. We could note that the WF of TiN in combination with Al_2_O_3_ is estimated to be about 4.53 eV and is in close agreement with the actual TiN WF reported in the literature [27,28]. What is surprising is the WF of Ru in combination with Al_2_O_3_, which is about 200–300 meV less than those reported in the literature for Ru metal [29,30]. It has been demonstrated, using internal photoemission experiments [31], that subtle changes in the chemical bonding at the metal/high-k interface can cause a significant impact on the barrier height (*Φ_b_*, as shown in Figure 2) at this interface. Such chemical modifications could occur from various processing, such as conditions of deposition, thermal budget, and ambient of annealing process. As a consequence, this could lead to a shift in the WF of the metal. However, it is possible to avert this interfacial reaction by using appropriate interfacial layer (IL), as can be seen from Figure 10. The WF of Ru improves to 4.8 eV by adding a thin (3 nm) TiN liner between Ru and Al_2_O_3_.

**Figure 11:**
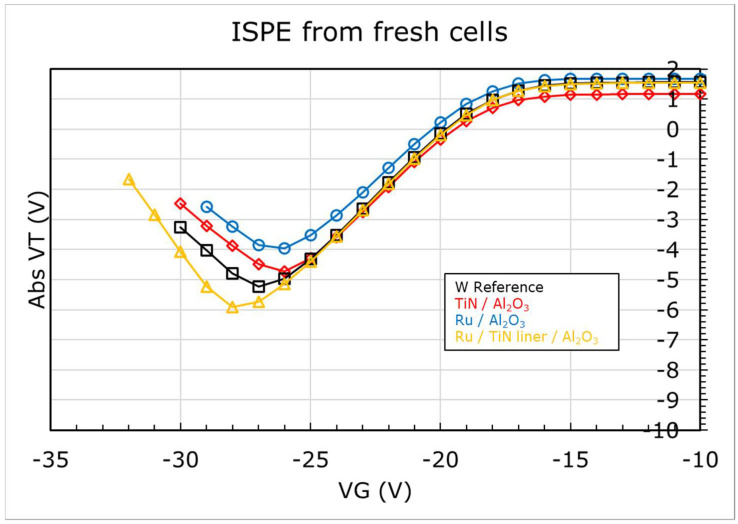
Incremental Step Pulse Erase (ISPE) of (Metal/High-k/ONO/Si) MHONOS, for different metal/high-k combinations from Figure 10.

In order to verify whether these shifts, measured in WF of Ru, reflect the actual change in metal WF, we compared the erase performance of these stacks. Figure 11 shows the ISPE curves for MHONOS stacks containing the metal/high-k combinations from Figure 10. The erase saturation (lowest VT shift achieved in ISPE) for TiN and Ru on Al_2_O_3_ (WF ~4.6 eV) is comparable after accounting for the differences in the starting *V_TH_*, while that of W Ref (WF ~4.9 eV) is better, corroborating the WF difference between these stacks. With the addition of TiN liner, the WF of Ru improves, and so does the erase saturation.

**Figure 12:**
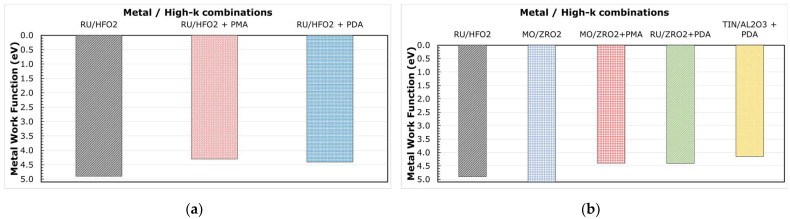
WF extracted for multiple metal and high-k combinations after different annealing conditions. (**a**) Ru with HfO_2_; (**b**) Ru, Mo with ZrO_2_, and TiN with Al_2_O_3_.

**Figure 13:**
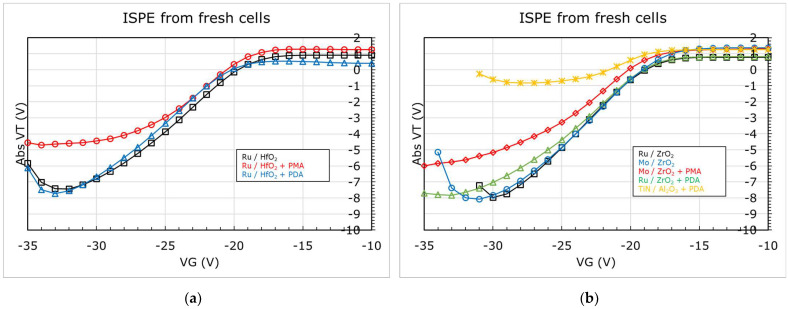
ISPE of MHONOS stacks for (**a**) Ru/HfO_2_. Erase performance degrades with post metallization anneal (PMA) while no change after a post high-k deposition anneal (PDA); (**b**) Ru and Mo with ZrO_2_ and TiN with Al_2_O_3_. Similar degradation after PMA as in the case with HfO_2_. However, worse performance with Al_2_O_3_.

We may note that the WF extracted from the Ru/TiN/Al_2_O_3_ stack is slightly less than that of W Ref, i.e., W/TiN/Al_2_O_3_ stack, yet the erase is better with Ru. Before addressing this, let us look at Figure 12a,b, which display the WF extracted for Ru, Mo, and TiN in combination with HfO_2_, ZrO_2_, and Al_2_O_3_ after different annealing conditions, as described in Figure 5. From Figure 12a, we could note a significant reduction (>500 meV) in the WF of Ru after the thermal treatment, irrespective of whether the metal electrode received the anneal (PMA) or not (PDA). The ISPE curves for these stacks are shown in Figure 13a. The stack that received the PDA does not change in erase while the one that received a PMA degrades both in erase slope and saturation level. We can also note from Figure 12b and Figure 13b that without any high temperature anneals, both Ru and Mo show similar WF and erase saturation levels in combination with ZrO_2_. Though after a thermal treatment (PMA or PDA), the WF reduces irrespective of the metal or high-k used, the erase saturation depends on the type of anneal applied. These observations (made from Figure 10, Figure 12, Figure 13) hint that (a) the WF alone is not the reason for erase functionality, and (b) an extra factor, unaccounted in the extraction, is affecting the WF, resulting in an effective work function, eWF, being measured from the experiments.

**Figure 14:**
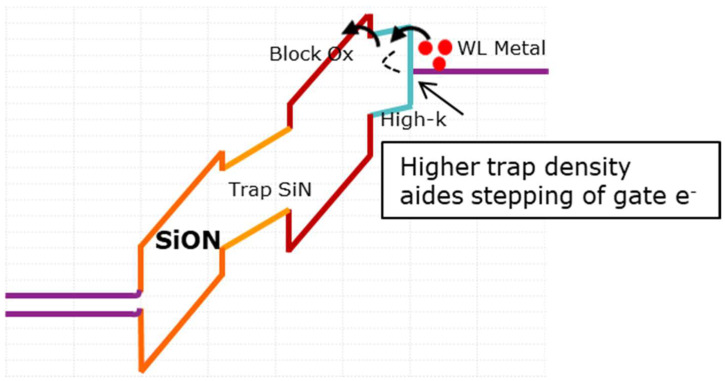
A typical band diagram of MHONOS during erase. Higher trap density reduces the tunneling path for gate electrons resulting in poor erase.

It is important to note that in the case of TiN with Al_2_O_3_ (PDA performed at 2min 1050 °C), the degradation in erase saturation is much worse, which is unlike the observations made for HfO_2_- and ZrO_2_-based stacks, and definitely not reflected in the WF reduction in TiN. A closer study on the high-k material properties reported elsewhere [32], investigated by trap spectroscopy, revealed that worse erase saturation levels at increased thermal budgets could be due to an increase in defect density in the high-k rather than a reduction in the metal WF itself. Higher defect density could increase trap-assisted tunneling [33], thereby increasing the leakage current during the erase operation (a typical band diagram during erase can be seen in Figure 14).

As discussed before, Fermi level pinning (FLP) at the metal/high-k interface, dipole formation at the high-k/SiO_2_ interface, and/or the energy offsets between the high-k and SiO_2_ have been suggested in the literature as possible root causes for an eWF. If the metal fermi level is pinned, then the *Φ_b_* at the interface should be different, which reflects in the erase saturation levels. Based on the observations made from Figure 11 for Ru with TiN liner and Figure 13 for Ru stacks after PDA, this effect can be ruled out. A common opinion in the literature [21,34,35,36] is that a dipole formed at the high-k/SiO_2_ interface is the dominant factor causing appreciable shifts in *V_FB_*, and hence, the WF extracted from it. Many physical models exist to explain this dipole formation, attributing it to dielectric contact induced gap states [37] or dictated by the electronegativity and ionic radii of the cations (from the high-k) [38], However, the most acceptable explanation seems to be oxygen vacancies driven by structural stabilization at the high-k/SiO_2_ interface [18,19,20,21,34,39,40]. Moreover, the dipole formation at the high-k/SiO_2_ interface should not affect the erase performance of flash memory, which is determined by the electron injection dynamics at the gate contact.

**Figure 15:**
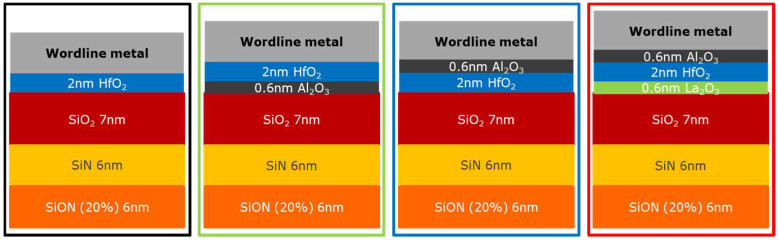
Schematic of MHONOS stacks with dipole-forming interlayers at different locations. Al_2_O_3_ and La_2_O_3_, each 0.6 nm thin, were used as interlayers with HfO_2_ used for high-k value.

**Figure 16:**
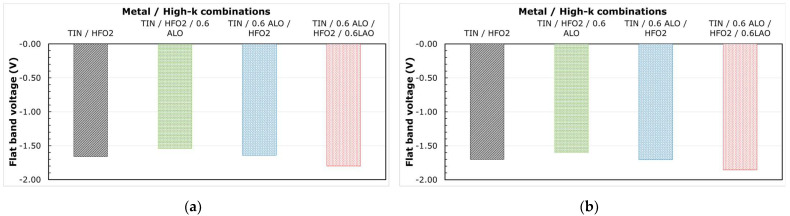
Flat band voltage monitored from CV traces, for MHONOS stacks with different dipole interlayers from Figure 15 (**a**) without any PMA and (**b**) with PMA for 20 min at 750 °C in N_2_ ambient.

To further clarify the impact of dipole formation on erase performance of flash memory, dipole-forming interlayers (DIL) [36,41,42], namely, Al_2_O_3_ and La_2_O_3_ (0.6 nm each), were studied as part of the MHONOS stack (shown in Figure 15). The DIL were deposited between metal and high-k or high-k and SiO_2_, with TiN/HfO_2_ being used as the control gate electrode and high-k dielectric. All the stacks received a PDA for 1.5 s at 1050 °C in N_2_ ambient. The corresponding shifts in *V_FB_* caused by the interlayers were extracted from CV measurements using CVC fitting (as can be seen in Figure 16).

We could note from Figure 16a that with the addition of Al_2_O_3_ DIL between HfO_2_ and SiO_2_, the *V_FB_* positively increases by about 120 meV, while it remains unchanged when Al_2_O_3_ is inserted between the metal and high-k. Though much higher *V_FB_* shifts are theoretically reported for Al_2_O_3_ [18], the processing conditions and thickness of the DIL play a major role in determining the magnitude of the *V_FB_* shifts [21,42,43]. Furthermore, if we add 0.6 nm La_2_O_3_ DIL between HfO_2_ and SiO_2_ while keeping the Al_2_O_3_ between TiN and HfO_2_, we notice a negative drop of about 140 meV in the *V_FB_*, which is in line with trends reported in the literature [44,45]. It is worth to note that the trend in *V_FB_* remains unchanged after a PMA for 20 min at 750 °C in N_2_ ambient (see Figure 16b).

**Figure 17:**
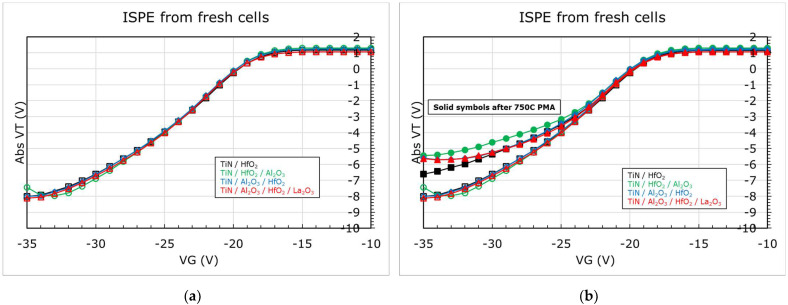
ISPE of MHONOS, for metal/high-k combinations from Figure 16. (**a**) Without any PMA; (**b**) with PMA for 20 min at 750 °C in N_2_ ambient.

The ISPE curves for these stacks without PMA are shown in Figure 17a. We could note, despite the differences in *V_FB_*, that there is no difference in the erase performance of these stacks. On the contrary, when the stacks were subjected to PMA, the erase depends on the material present in the stack, as can be seen in Figure 17b. The control sample with only TiN and HfO_2_ shows slight degradation after PMA. However, the stacks with DIL show higher reduction in erase, even worse when the Al_2_O_3_ is present next to the blocking oxide, though it shows a positive *V_FB_* shift (indicating a higher eWF). It is well known that Al_2_O_3_ dielectric suffers from a wider band of defect profile [46]. Recalling the discussion from before on the possible impact of defect density in the high-k on erase (see Figure 14), we could fairly say that the above results corroborate this hypothesis.

## 4. Conclusions

We have extracted and studied the shifts in metal work function (i.e., effective work function, eWF), in response to different processing parameters, such as gate electrode and high-k dielectric materials, and variations in annealing conditions. By studying the work function in combination with the erase performance of NAND flash memory, we were able to narrow down the origin of eWF to dipole formation due to (a) interfacial reactions at the metal/high-k interface and/or (b) possible oxygen vacancies driven by structural stabilization at the high-k/SiO_2_ interface. It must be noted that based on the above studies, we did not observe fermi level pinning at the metal/high-k interface.

We also verified and validated the negligible impact of dipole on erase performance by studying different dipole forming interlayers in the memory cell. It is clear that the metal WF extraction is convoluted by dipole formation, while the erase performance of a flash memory cell is affected more by the trap profile in the high-k liner than any other factors that cause shift in flat band voltage.

## Figures and Tables

**Figure A1:**
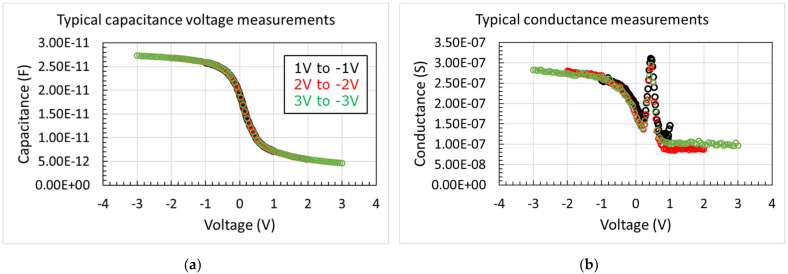
Typical (**a**) capacitance and (**b**) conductance measurements performed in this work. Data shown for Ru/HfO2 combination on a SiO_2_ bevel (slant etch) on a p-type Si substrate. The capacitors are sequentially measured at different voltage sweep ranges (i) 1 V to −1 V, (ii) 2 V to −2 V, (iii) 3 V to −3 V.

**Figure A2:**
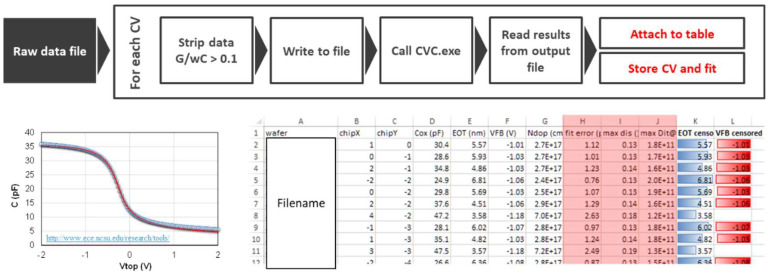
Example procedure of data extraction from measurement.

## Data Availability

Not applicable.

## Appendix A

Figure A1 shows the typical capacitance and conductance curves obtained on 70 × 70 μm^2^ capacitors at a frequency of 100 kHz. The capacitors were fabricated on a p-doped, 300 mm Si substrate with SiO_2_ bevel. Data is shown for 3 nm HfO_2_ high-k liner and Ru gate electrode. The capacitors are sequentially measured at different voltage sweep ranges (i) 1 V to −1 V, (ii) 2 V to −2 V, (iii) 3 V to −3 V. We could notice that there is little impact of the voltage sweep on the hysteresis of the curves. Capacitance data from the 2 V to −2 V voltage sweep range is then used to subsequently extract the flat band voltage, *V_FB_*.

Figure A2 shows the schematic of an automated *V_FB_* extraction with a robust and traceable procedure. Test for gate leakage is performed in the measurement routine (not shown) and warnings are issued if any issues are encountered. Only those data with appropriate fit errors are filtered for further analysis. The rest of the analysis follows as discussed in the main article (see page 5 onwards).
